# Calcium Phosphate Cements as Carriers of Functional Substances for the Treatment of Bone Tissue

**DOI:** 10.3390/ma16114017

**Published:** 2023-05-27

**Authors:** Yulia Lukina, Tatiana Safronova, Dmitriiy Smolentsev, Otabek Toshev

**Affiliations:** 1 National Medical Research Center for Traumatology and Orthopedics Named after N.N. Priorov, Ministry of Health of the Russian Federation, Priorova 10, 127299 Moscow, Russia; smolentsevdv@cito-priorov.ru; 2 Faculty of Digital Technologies and Chemical Engineering, Mendeleev University of Chemical Technology of Russia, Miusskaya pl. 9, 125047 Moscow, Russia; 3 Department of Chemistry, Lomonosov Moscow State University, Building 3, Leninskie Gory 1, 119991 Moscow, Russia; safronovatv@my.msu.ru; 4 Department of Materials Science, Lomonosov Moscow State University, Building 73, Leninskie Gory 1, 119991 Moscow, Russia; otabektoshev0995@mail.ru

**Keywords:** calcium phosphate cement, hydroxyapatite, brushite, functionalization, bone tissue, treatment, osteomyelitis, antibiotics, growth factors, antitumor drugs

## Abstract

Interest in calcium phosphate cements as materials for the restoration and treatment of bone tissue defects is still high. Despite commercialization and use in the clinic, the calcium phosphate cements have great potential for development. Existing approaches to the production of calcium phosphate cements as drugs are analyzed. A description of the pathogenesis of the main diseases of bone tissue (trauma, osteomyelitis, osteoporosis and tumor) and effective common treatment strategies are presented in the review. An analysis of the modern understanding of the complex action of the cement matrix and the additives and drugs distributed in it in relation to the successful treatment of bone defects is given. The mechanisms of biological action of functional substances determine the effectiveness of use in certain clinical cases. An important direction of using calcium phosphate cements as a carrier of functional substances is the volumetric incorporation of anti-inflammatory, antitumor, antiresorptive and osteogenic functional substances. The main functionalization requirement for carrier materials is prolonged elution. Various release factors related to the matrix, functional substances and elution conditions are considered in the work. It is shown that cements are a complex system. Changing one of the many initial parameters in a wide range changes the final characteristics of the matrix and, accordingly, the kinetics. The main approaches to the effective functionalization of calcium phosphate cements are considered in the review.

## 1. Introduction

Bone tissue is a part of the human musculoskeletal system, which participates in the transfer of force from one part of the body to another under controlled tension, and protects and fixes internal organs. In addition to performing a mechanical function, bone tissue performs a biological function, as it participates in metabolism [1,2,3,4]. Bone tissue is a reservoir of calcium and phosphate ions in the form of hydroxyapatite, so it plays an important role maintaining the proper calcium levels, along with in other organs [5,6].

Bone tissue’s ability to regenerate effectively, maintain mineralization and repair itself after damage depends on its ability to dynamically remodel. However, the regenerative process is limited by the ability to self-repair: osteogenic insufficiency occurs if the critical size of the defect is exceeded, and the defect is filled with fibrous connective tissue.

There are many different clinical circumstances under which a significant part of bone or a whole bone is lost. Bone defects can be caused by various reasons. They can be associated with various pathogenic conditions and clinical outcomes, including injuries (fractures), infections (osteomyelitis), tumors, osteoporosis, and many other bone diseases [7].

According to statistics, 20 million orthopedic surgeries in the world per year are performed, 70% of which require the use of bone implant material for filling and repairing bone defects [8].

Various osteoplastic materials can be used to fill in bone defects caused by various diseases, or for the purpose of their prevention. They are able to deliver functional substances locally, fill in defects and serve as a material for bone tissue reconstruction.

Calcium phosphate cements are similar in composition to the mineral component of bone tissue. They have a high specific surface area and are used in medicine as osteoplastic materials. Blocks and granules made of pre-hardened cement are a promising type of skeleton for the restoration of bone defects. They have an increased rate of resorption compared to matrices obtained by high-temperature processing. Functional substances can be volumetrically incorporated into them at the stage of mixing the components. The kinetics of the release of functional substances may vary. The release of Ca^2+^ ions during resorption can affect the differentiation of osteogenic cells and the level of inflammatory cytokines.

The functionalization of calcium phosphate cements is of great clinical interest for the treatment or prevention of various diseases of bone tissue. Prolonged elution is the main requirement for carrier materials of pharmaceutical substances. Another important requirement is the absence of a mutual negative influence of the functional substance and the matrix on each other’s properties. The release of functional substances from calcium phosphate cements depends on many parameters of the matrix, specific interactions between the functional substance and the matrix, as well as environmental factors.

A significant advantage of calcium phosphate cements is the wide range of changes in the properties of matrices that can affect the release of functional substances and the process of bone tissue restoration.

## 2. Composition and Structure of Bone Tissue

Bone is a highly organized composite material consisting of 50–70% inorganic components (mainly hydroxyapatite), 20–40% organic components, 5–10% water and 3% lipids [9]. That is, the structure of bone tissue is a composite of reinforcing and matrix phases. The reinforcing phase mainly consists of hydroxyapatite crystals. It provides strength and rigidity, while the matrix phase mainly consists of collagen fibrils. They provide flexibility and elasticity to the bone [10]. The organic part consists mainly of type I collagen, 10% non-collagen proteins, lipids, proteoglycan molecules, osteopontin, osteonectin, osteocalcin, sialoproteins, morphogenetic proteins and phosphoproteins. There are more than 200 types of non-collagen proteins; among them, 12 species predominate [10]. Bone matrix proteins also play a vital role in the mechanical strength and adhesive characteristics of tissues. Osteoblasts synthesize the organic substances of the bone matrix. The mineralization of osteoids (non-mineralized organic matrix) occurs due to the appearance of matrix vesicles in the osteoid, secreted by osteoblasts. Vesicles contain Ca^2+^ ions and phosphatases, and form amorphous calcium phosphate on the surface, followed by the formation of hydroxyapatite crystals from it. Hydroxyapatite is the most important inorganic phase in bone (molecular formula Ca_10_(PO_4_)_6_(OH)_2_; it contains impurity ions such as CO_3_^2−^, Cl^−^, F^−^, Na^+^, Mg^2+^, K^+^, Zn^2+^, Fe^2+^, Cu^2+^, Sr^2+^ and Pb^2+^ [8].

In addition, various other mineral phases, such as amorphous calcium phosphate, monocalcium phosphate, and dicalcium phosphate dehydrate, are present in bones [8]. Carbonate groups, lining up in the structure of hydroxyapatite at 2–8%, form a phase of carbonate-containing non-stoichiometric hydroxyapatite, with the general formula Ca_10−x−y/2_(HPO_4_)_x_(CO_3_)_y_(PO_4_)_6−x−y_(OH)_2−x_ [11,12].

Bone tissue has a complex multiphase, heterogeneous, anisotropic microstructure [1]. Due to its heterogeneity and anisotropy, the hierarchical structure of natural bone is divided into several different levels, from nano and macro to the level of whole bone [11,13]. In the literature, there is a division into four, seven or nine levels [10,13,14,15]. The unevenness of the bone structure largely depends on the number of levels in the assessment scale and the level used for analysis.

The bone is mainly formed by the external cortical bone and the internal spongy bone at the macroscopic or mesoscopic levels [10]. Compact and spongy macroscopic structures have a smooth structural transition inside the bone and make up 80% and 20% of the total bone mass, respectively [16]. The main function of the cortical bone, as a dense bone tissue, is to stabilize and support the internal porous structure. Spongy trabecular bone provides a favorable environment for tissue metabolism and hematopoiesis of the bone system [17,18].

The mechanical properties of the cortical bone at the macro level are closely related to its microstructure and composition. Moduli of elasticity and strength decrease with increasing porosity or area of osteons. Changes in the mineral content, for example, as a result of aging, and the accumulation of microcracks due to a decrease in remodeling activity are factors affecting the physico-mechanical properties of the cortical bone [19].

Compact and spongy bones have different mechanical properties due to the different contents of inorganic and organic substances [20,21]. A large number of ex vivo studies have reported values of elasticity of the cortical bone layer [22]. The average Young’s modulus along the diaphysis is about 14–20 GPa [19,23], while perpendicular to the diaphysis it is about 11 GPa [24]. The average shear modulus corresponding to the torsion experiment around an axis parallel to the diaphysis is about 4–6 GPa [20,24]. The compressive strength of the cortical bone along the diaphysis is 188–222 MPa, while perpendicular to the diaphysis it is 110–152 MPa, and the bending strength is 119–151 MPa and 42–64 MPa, respectively [8]. The strength of the trabecular bone is 1–12 MPa, with an elastic modulus of 0.022–0.702 GPa. The strengths of vertebrae and tubular bones differ significantly [8].

## 3. Causes of Bone Tissue Damage and Ways of Treatment

### 3.1. Injury

Bone injuries caused by trauma can occur in patients of all ages. This can be the result of traffic accidents, falls, and many other reasons. Bone fractures are one of the most common types of injuries.

Injuries caused by trauma can be divided into long bones and spine, or maxillofacial and craniofacial, depending on their localization. The most common places of bone fractures are the femur, shoulder (mainly humerus), hip (femoral neck), wrist (radius/ulna), tibia (distal third), ankle, vertebra, and maxillofacial and craniofacial areas (jaw bone, cranial vault) [25].

The mechanism of bone repair is a multi-stage organized restorative procedure, involving a number of vital progenitor cells along with inflammatory, endothelial and hematopoietic cells [26,27]. Cortical tissue, periosteum, bone marrow and external soft tissues contribute to the healing process. It depends on many parameters present in the damaged tissue, such as growth factors, hormones and nutrients, pH, oxygen saturation, and the performed mechanical stabilization of the fracture [28].

**Figure 1:**
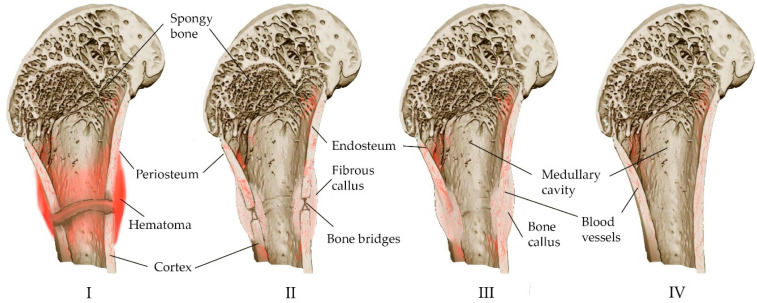
Schematic representation of the stages of fracture self-healing: (**I**) The acute stage of the fracture, a hematoma forms around the damaged area of the bone, the bone tissue of the ends of the fragments partially dies (colored dark), local enzymatic activity increases. (**II**) Development of connective tissue corn. Accompanied by inflammation: cells participating in the inflammatory response appear at the site of the fracture; osteoclasts and osteoblasts are active, non-viable tissue is processed; with the formation of a “cloud” of new tissue at the site of the fracture, structured bone bridges appear inside the corn connecting the fragments. (**III**) Consolidation: the newly formed tissue acquires the correct bone structure, and trabeculae appear. (**IV**) Remodeling: the newly formed bone acquires its final structure.

The bone restoration procedure consists of several overlapping phases [8,26,29], which can be combined into three main phases: inflammation, bone formation, and bone modeling (Figure 1).

Inflammation begins immediately after a bone fracture and lasts for several days. In the fracture area, blood vessels are damaged, bleeding occurs, hematoma forms caused by osteocyte necrosis due to hypoxia, and thrombosis ensues with the formation of fibrin mesh along the fracture line [8,25,26,30,31,32]. The fibrin mesh isolates the fracture site and serves as a framework for the infiltration of inflammatory cells and macrophages, neutrophils and mast cells that release cytokines and growth factors. Macrophages that have migrated to the site of inflammation remove the temporary fibrin matrix, necrotic cells and bone fragments [25]. As a result, the hematoma and acute inflammatory reactions disappear after a week.

The release of cytokines and growth factors together with pro-inflammatory stimuli leads to a high production of prostaglandins [28]. Newly formed capillaries from the periosteum, and fibroblasts and bone formations of an invasive hematoma appear. Fibroblasts secrete a large number of collagen fibers, and after differentiation, the hematoma forms a fibrous callus, which is characterized by neovascularization, migration of mesenchymal cells, and fibroblast ingrowth [8,31]. Neovasculogenesis in combination with the further production of growth factor and prostaglandins contributes to the differentiation of mesenchymal stem cells towards chondrogenic or osteogenic, and the initial formation of bone tissue [8,33].

The process of the transformation of fibrous callus into bone callus occurs at 2–3 weeks, mainly due to the differentiation of progenitor cells into osteogenic cells (chondrocytes and osteoblasts), secreting matrix osteoids, which gradually replace the fibrous callus due to the deposition of calcium salts (mineralization) [8,32]. It is believed that the relative distance of cells to blood vessels is one of the factors in the differentiation of progenitor cells into osteoblasts, along with the release profiles of cytokines and growth factors. The distance must be kept to a minimum because osteoblasts depend on oxidative metabolism, and require a constant and substantial supply of oxygen and nutrients. Therefore, they accumulate in the immediate vicinity of the newly formed blood vessels. The adapted metabolism of chondrocytes is designed to survive and function in a poorly vascularized environment, so they mature farther from the blood vessels [25].

Chondrocytes proliferate, forming a cartilaginous callus, and then turn into a bone callus via the internal osteogenesis of cartilage [8]. Gradually, the soft callus is replaced by a hard callus at 3–4 months [32], which is visible on radiographs [26].

Bone remodeling, the final stage of healing, lasts for more than several months. In the process of regeneration, the bone regenerates and returns to its original shape [26,28], and the callus is rebuilt according to the needs of biomechanics. Osteoclasts absorb excess callus and recanalize the bone marrow cavity. Insufficient bone callus is replenished due to membrane osteogenesis [8].

Trabecular bone repair occurs without significant external callus formation. After the inflammatory stage, intramembranous ossification predominates in bone formation. This is explained by a significant angiogenic response [32].

The healing of bone fractures is a complex regenerative process. Pathological conditions (the presence of cracks, impaired blood flow, concomitant infection and extensive damage to soft tissues), insufficient mechanical stability and metabolic disorders (diabetes, age-related osteoporosis, genetic factors) are inhibitory factors [33,34]. Up to 10% of people experience delayed healing or bone non-fusion. Biomechanical stability is a critical factor in the healing process of fractures. Internal or external fixation is intended to improve stability and promote healing [35]. The stability of the fracture is of paramount importance to the prevention and treatment of fracture-associated infection [32,36]. Antibiotic-loaded spacers are used in the staged reconstruction of bone non-fusion and bone defects [37].

Poor bone quality in patients with osteoporosis and diabetes can lead to a more complex fracture structure and problems with fixation [7]. The shell of soft tissues can be disrupted due to chronic vascular disease, the prolonged use of steroids, and a decrease in skin turgor. This leads to open injuries, even with low-energy fractures [38].

The use of exogenous bioactive factors at the site of a defect to accelerate bone repair has been under extensive investigation in the field of bone regeneration. Differences in structure determine their different physiological roles and their special role in ensuring the growth of new bones [27,39,40,41]. Vascular endothelial growth factor (VEGF), insulin-like growth factors (IGFs), fibroblast growth factors (FGFs), bone morphogenetic proteins (BMPs), platelet-derived growth factor (PDGF), and transforming growth factors (TGFs) are the most widely studied in areas of bone regeneration at present. Effective results of regeneration in bone defects depend on the growth factor delivery system.

The concept of using bioactive agents such as tetracycline (antibiotic) and flurbiprofen (non-steroidal anti-inflammatory drug—NSAID) and their chemically modified analogues for early bone formation has recently been proposed in bone regeneration studies [39]. They are effective antiresorptive agents, interfere with intracellular calcium concentration and act as strong inhibitors of osteoclasts. However, there are conflicting data on the effectiveness of the use of NSAIDs. They lead to a delay in healing and a decrease in the content of minerals and matrix of the callus, inhibit remodeling, and cause non-fusion, according to the results of some studies [33,42], and have shown almost no effect on fracture healing in other studies [43]. NSAIDs are useful in everyday clinical practice for pain relief due to their pronounced analgesic activity and anti-inflammatory effects, but conflicting preclinical results, as well as the lack of well-randomized clinical data, suggest that more research is needed [33].

Bisphosphonates and anabolic agents inhibit bone resorption and can be used to accelerate bone tissue repair [44]. Despite the possible stoppage of remodeling and an increase in bone fragility [45], the negative consequences do not outweigh their beneficial effect at the moment; for example, in the prevention of additional fractures in patients with osteoporosis, who are often diagnosed after primary fractures [33].

### 3.2. Osteomyelitis

Osteomyelitis is an inflammatory bone disease caused by an infectious microorganism, often accompanied by bone destruction. It is most often caused by the local spread of infection after injuries, orthopedic operations or joint replacement. The disease may be limited to a specific area of bone or several areas, such as the bone marrow, cortical, periosteum and surrounding soft tissues [34,46,47]. Different types of osteomyelitis require different medical and surgical therapeutic strategies. The most common osteomyelitis is secondary to the site of infection after injury, surgery, or the installation of an articular prosthesis. Osteomyelitis secondary to vascular insufficiency (diabetic foot) or hematogenic origin is less common.

The main causative agents of bone infections are Gram-positive cocci, including Staphylococcus aureus, coagulase-negative staphylococci (*Staphylococcus epidermidis*), enterococci (*Enterococcus faecalis*) and streptococci, and Gram-negative anaerobic bacteria (*Escherichia coli, Pseudomonas aeruginosa*) [48,49,50,51]. *S. aureus* has a high level of antibiotic resistance, which has long been recognized. Many other microorganisms, including *S. epidermidis* [52] and a number of staphylococci, have demonstrated increasing antibiotic resistance in recent years [53]. It has been reported that up to 40% of *S. epidermidis* strains [54] and 32% of *S. aureus* strains [55] isolated from orthopedic postoperative and implant-related infections are resistant to gentamicin.

Methicillin-resistant *S. aureus* (MRSA) is considered particularly virulent due to the production and release of a number of extracellular and cell-associated factors [48,56,57]: bacterial adhesins (microbial surface components that recognize and specifically interact with a single host protein component such as fibrinogen, fibronectin, collagen, and others), toxins, capsular polysaccharides (avoiding host defenses), exotoxins, and various hydrolases (invasion or penetration into tissues by a specific attack on host cells or degradation of extracellular matrix components). *S. aureus* internalized by cultured osteoblasts can survive inside cells [57,58]. As soon as *S. aureus* enters the cell, the activity and viability of osteoblasts decrease, and the expression of an apoptosis-inducing ligand associated with tumor necrosis factor is induced [48]. Infected osteoblasts secrete cytokines, chemokines, and growth factors. They are involved in the immune response and promote bone destruction, bacterial adhesion, and biofilm formation [48,59]. A biofilm is a microbial community and consists of cells that adhere to a substrate, an interface, or to each other. They are embedded in an extracellular polymer matrix and exhibit an altered phenotype in terms of growth, gene expression, and protein production [57,60]. Biofilm complicates treatment by acting as an impenetrable barrier that prevents the penetration of antibiotics and immune cells.

The strategy for the treatment of osteomyelitis is based on the use of antibiotics systemically and/or locally, alone or in combination with debridement. Many factors, including patient-specific factors, microorganism type and antibiotic susceptibility, location, spread, implant loosening, and most importantly, the type of infection determined by the time of onset (acute or chronic), affect the overall treatment algorithm [61,62].

In periprosthetic infections, methicillin-resistant *S. aureus* (MRSA) predominantly causes early infections, whereas methicillin-sensitive *S. aureus* (MSSA) causes delayed and late infections [49].

**Figure 2:**
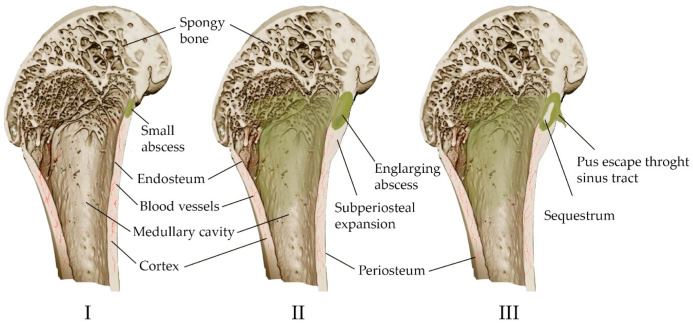
Scheme of the development of hematogenous osteomyelitis in the bone. (**I**) Formation of a primary bone abscess (necrosis and purulent fusion of the bone marrow and adjacent bone, limited by the walls of healthy tissue). (**II**) Subperiosteal abscess (pus through the haversian canals of the bone spreads under the periosteum, exfoliating it from the bone). (**III**) Fistula formation (pus breaks out through soft tissues); the formation of sequesters is possible—areas of the bone completely devoid of vascular nutrition and lying freely.

Acute purulent inflammation is characteristic of acute osteomyelitis (Figure 2). Various inflammatory factors and leukocytes contribute to tissue necrosis and destruction of bone trabeculae and bone matrix. Vascular channels are compressed and obliterated by the inflammatory process. The ischemia resulting from the process also contributes to bone necrosis. Segments of bone that are deprived of blood supply may separate as sequesters and continue to contain bacteria despite antibiotic treatment [57]. However, antibiotic therapy alone is often sufficient to treat acute osteomyelitis [34]. The high activity of osteoclasts causes bone loss and localized osteoporosis. Meanwhile, the bones converge, in some cases excessively, causing the periosteum to converge and new bone to form [57].

Chronic osteomyelitis is still difficult to treat, and has significant morbidity and a high risk of recurrence. It is associated with avascular bone necrosis and sequestration. The infection usually does not begin to regress until the site of persistent contamination has been surgically removed. Antibiotic therapy alone is usually not enough to treat chronic osteomyelitis, although antibiotics relieve many symptoms [34,63]. Rare complications of chronic osteomyelitis include squamous cell carcinoma at the site of tissue drainage and amyloidosis [64].

Systemic antibacterial therapy becomes ineffective in the presence of a vascular area or poorly vascularized scar tissue in osteomyelitis, since antibiotics cannot reach infected tissue [57,61].

In such cases, there is a significant need to develop matrices with antibacterial properties.

Thus, the specific etiology of bone defects caused by inflammation imposes many requirements on the design of medical materials, including the ability to cope with both the inhibition of inflammatory responses and the stimulation of bone tissue regeneration [34,61]. Several principles for topical antibiotic treatment are particularly important: maintaining a concentration above the minimum inhibitory concentration (MIC) of antibiotics that affects bacteria for at least three to six weeks, and adequate tissue penetration so as not to cause local and systemic toxicity. High concentrations of antibiotics (up to 1000-fold increase) are needed to destroy the introduced microorganisms in the case of a formed biofilm.

One of the main advantages of the biodegradable system is the absence of secondary surgical procedures to remove foreign material after the release of antibiotics, such as PMMA cement, has ceased. Additional possibilities for using resorbable systems include changing the release of antibiotics, and the possibility of targeted adjustment of the wound environment by the products of material degradation [65]. Resorption must be complete to leave no substrate for bacterial colonization and to promote the integration of the host tissue. The kinetics of elution of an antibiotic from a material are closely related to its characteristics, provided by composition, surface area, porosity, and other factors (Hanssen, 2005).

The most commonly used antibiotics are gentamicin, rifampicin, vancomycin and tobramycin [62].

It is necessary to take into account the local concentration of antibiotics when they are applied topically, since high concentrations of antibacterial drugs have a cytotoxic effect on cell viability, cause osteogenic differentiation, and reduce the expression levels of genes and the proteins of collagen [66].

### 3.3. Tumor

**Figure 3:**
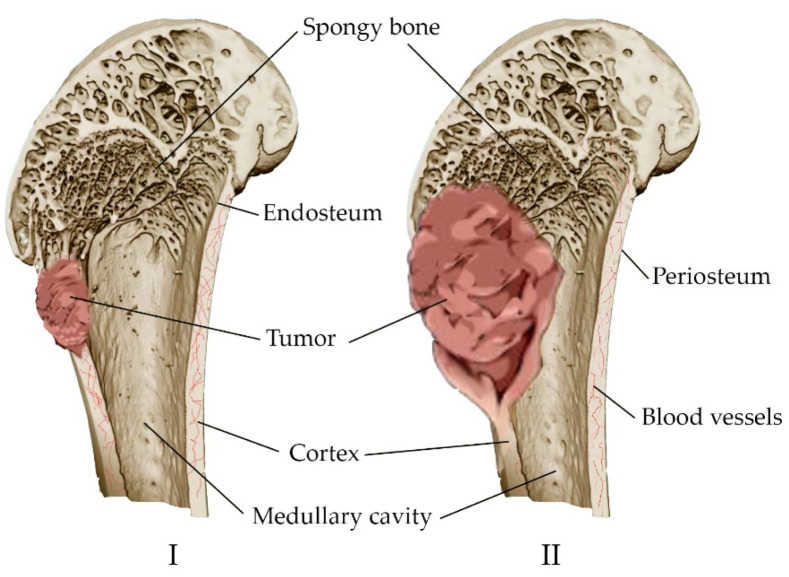
Development of a bone tumor: (**I**) Destruction of a bone site by a tumor; (**II**) replacement of healthy bone tissue with tumor tissue, inclusion of the bone marrow canal and soft tissues in the pathological process.

The main concept of orthopedic oncology is the prevention of amputation. It is performed in 10–20% of patients with malignant bone tumors [67]. The tumor is characterized by the replacement of healthy bone tissue with tumor tissue, with the inclusion of the medullary canal and the soft tissues surrounding the bone in the pathological process (Figure 3). Many tumors originate in the metaphyseal–diaphyseal regions of long bones and can be segmentally resected while preserving the joint [68]. Approaches have been developed for the treatment of complications associated with malignant neoplasms. These include either anticancer intervention, such as chemotherapy, hormone therapy, radiation therapy, and resection, or bone-supportive care, such as calcium, analgesics, and vitamin D supplements [69].

Metastatic bone lesions are common in patients with other types of cancer (lung, breast, prostate, and colorectal cancer). Most severe bone metastases are also treated with reconstructive surgery. This is followed in some cases by postoperative radiation or chemotherapy. Patients after body reconstruction still show a risk of developing severe complications such as tumor recurrence [70].

Depending on the type of cancer, metastatic lesions can be characterized as osteolytic, osteoblastic or mixed lesions (containing both elements), in which the regulation of the normal process of bone remodeling is disrupted [71]. Osteolytic lesions are characterized by the leaching of the mineral part of the bone, its thinning, and fractures. Osteoblastic metastases, on the contrary, are characterized by compaction of the mineral part of the bone, since the cells of different tumors can both directly destroy bone tissue and stimulate cells that renew it. Osteolysis is also observed in prostate cancer, despite the tendency to osteoblasting in metastases. However, there is a shift in the balance towards the formation of a new matrix and its mineralization. The increase in bone volume is due to the replacement of existing trabecular tissue with abnormally woven bone, which creates a general appearance of sclerosis [72].

Osteolytic metastases can cause severe pain, pathological fractures, life-threatening hypercalcemia due to elevated blood calcium levels, spinal cord compression, and other nerve compression syndromes [73]. Patients with osteoblastic metastases feel pain in bones and pathological fractures due to poor bone quality produced by osteoblasts [71].

Drugs that block bone resorption may reduce bone pain and the risk of pathological fractures in metastatic lesions. One of the effective groups of drugs used in osteolytic bone disease and osteoblastic bone disease associated with prostate cancer metastasis is bisphosphonates [69,71,72,74,75]. Bisphosphonates have the ability to inhibit resorption processes in the bone by reducing the activity of osteoclasts. Bisphosphonates inhibit the release of growth factors, inhibiting bone resorption, and thus block the feedback from tumor cells. This helps to reduce the activity of tumor cell proliferation. Bisphosphonates can induce apoptosis in malignant cells similar to the process observed in osteoclasts, and reduce the adhesion of tumor cells to bone, reducing the risk of new metastatic lesions.

If there is a high probability of a pathological fracture of metastatic bone tissue, prophylactic fixation is recommended [76]. This involves the resection of the tumor and reconstruction of the damaged bone [77]. Plates, intramedullary rods and screws are used as fixators in the reconstruction of a damaged bone. With lesions of more than 50% of the bone diameter or joint lesions, prostheses are installed [78,79]. The purpose of reconstruction is palliative. It reduces pain and restores the function of the affected bone throughout the life of the patient. Among the complications of reconstructive treatment of metastatic lesions of the bone tissue are infection (0–11.7%), aseptic loosening (0–12.5%), mechanical damage (0–14.7%), and tumor recurrence (3.1–14.7%), despite the fact that chemotherapy is prescribed after reconstruction [70,80,81,82,83].

The combination of high doses of methotrexate, cisplatin, ifosfamide, and doxorubicin results in increased survival, but the use of anticancer drugs is limited by serious side effects. Poor bone blood supply, drug resistance, and nonspecific absorption require the use of highly toxic doses of anticancer drugs [74]. Therefore, there is a need to develop locally delivered carrier materials with reduced side effects and/or without effects for the treatment and prevention of growth-related bone cancers.

Surgical resection together with radiation/chemotherapy is a clinically accepted treatment regimen. For biomaterial therapy, surgical intervention is necessary to remove tissue and to provide space for the formation and integration of new bone. Biomaterials are used as bone substitutes after tumor surgery. Adjuvant treatments, such as systemic radiation therapy and chemotherapy, are used to prevent relapse.

The localization of radio/chemotherapy is a more highly effective method of the treatment and prevention of relapse after tissue reconstruction, since simultaneous processes of tumor inhibition and bone regeneration are possible with the mobilization of drugs via bone replacement material [84].

Many surgeons use cement reconstruction techniques to minimize postoperative complications. Bone cement made of polymethylmethacrylate is used as a carrier of chemotherapeutic drugs in an attempt to reduce tumor recurrence, and serves as an addition to bone reconstruction [70].

At the same time, inorganic calcium phosphate cements attract the attention of researchers because their biological properties can counteract the toxic nature of the inclusion of anticancer drugs, and promote osteogenesis. The presence of calcium phosphate cement can help to avoid the spread of tumor cells, and prevent the development of new lesions in the surrounding tissues during resection inside the focus [85]. They can also be used as carriers of antitumor drugs or radioactive substances, and administered to patients to achieve antitumor effects [86,87]. These cement modifications for the delayed release of antitumor drugs, magnetic tumor targeting, or radiological modifications are designed to simplify the treatment process and reduce systemic side effects and pain in patients [88].

### 3.4. Osteoporosis

**Figure 4:**
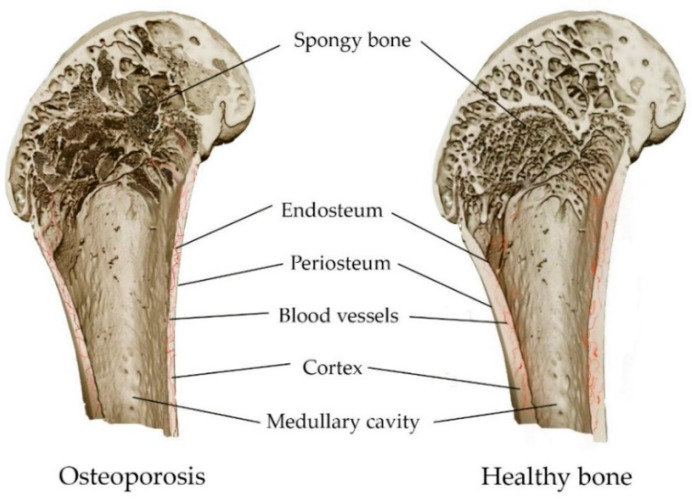
Comparison of osteoporosis and healthy bone: decrease in bone density, and thinning of bone structures, as a result of osteoporosis.

An imbalance in bone metabolism between bone resorption mediated by osteoclasts and bone formation mediated by osteoblasts leads to the occurrence of metabolic diseases in bones, including osteoporosis and osteomalacia (mineralization deficiency caused by a a lack of calcium or phosphorus, or insufficient activity of osteoblasts) [89,90]. Osteoporosis is a leading public health problem and one of the most common chronic diseases. It affects more than 200 million people worldwide [91]. A systemic metabolic disorder in the bones leads to a gradual loss of bone mass and damage to microarchitectonics, along with the weakening of bone strength (Figure 4). This leads to low-energy fractures [34,92]. A fracture can occur anywhere in the skeleton, although fractures of the wrist, hip and spine are the most common [89]. Both men and women lose bone mass as they age, with women gradually losing 50% of trabecular and 30% of cortical bone over a lifetime, while men lose two-thirds of this amount [93]. The prevention of osteoporosis can be achieved via a balanced diet containing calcium, phosphorus and vitamin D. These improve bone reabsorption and repair, except in cases of hereditary bone diseases [89,94].

The pathogenesis of osteoporosis is mainly associated with bone homeostasis, with the balance of bone remodeling between formation and resorption by specific cells, including osteoblasts, osteocytes and osteoclasts. Mesenchymal stem cells of the bone marrow are multipotent cells with the ability to differentiate into lines of osteoblasts, chondrocytes and adipocytes [95]. Osteoporosis is an increase in the adipose tissue of the bone marrow due to a shift in differentiation into adipocytes rather than osteoblasts. In addition, activation of the main signaling pathways of bone metabolism can both promote the differentiation of pre-osteoclasts into osteoclasts, and prevent it by suppressing the RANKL membrane protein [34].

A common and effective strategy for the treatment of osteoporosis is antiresorptive therapy, which targets osteoclasts and reduces the rate of bone resorption [34,74,92,96]. The therapeutic efficacy of bisphosphonates and monoclonal antibodies has been confirmed by the successful use of pharmaceutical preparations alendronate, risedronate, zoledronate, raloxifene ibandronate, teriparatide, abaloparatide and denosumab [74,89,92,97]. Bisphosphonates can minimize the chances of vertebral fractures by 50–60% and hip fractures by 50% [98]. Anabolic agents (romosozumab, teriparatide, and abaloparatide) are currently approved for the treatment of osteoporosis because the drugs may promote bone regeneration and reduce bone fractures [89,97].

In addition, osteoblasts are able to respond to various modalities of the extracellular signal, including the concentration of extracellular free ionized calcium Ca^2+^, regardless of systemic factors [99].

Local prolonged drug delivery systems in effective therapeutic doses to the site of bone disease can contribute to the effective treatment of various metabolic diseases of the bone tissue, with less adverse effects [89,100,101]. Minimal invasiveness can be considered as a concept to reduce the risk and number of complications [34].

**Figure 5:**
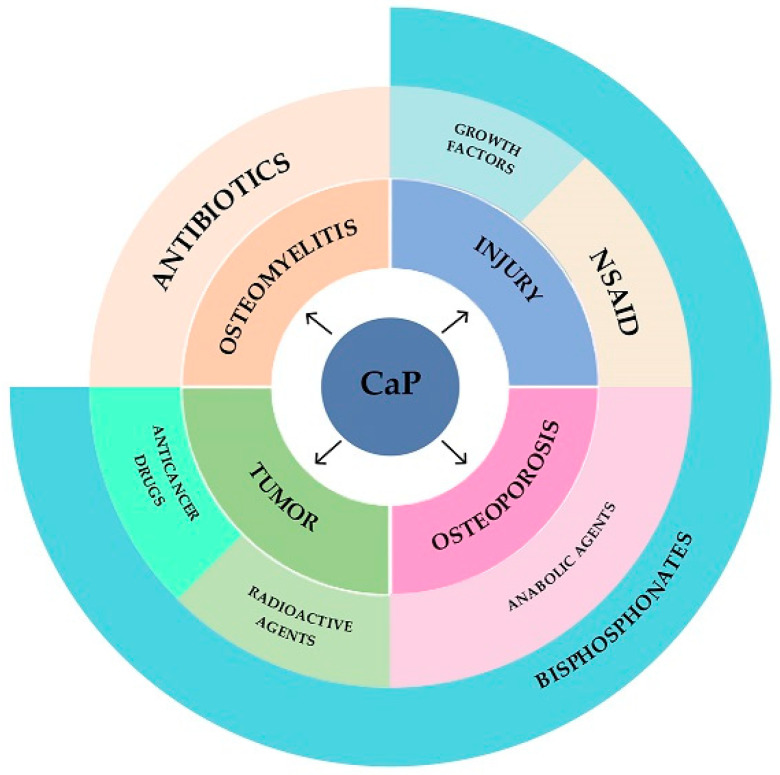
Diagram of applicability of calcium phosphate cements.

Thus, calcium phosphate cements can be used as carriers of antitumor, antibacterial, radioactive, anabolic, antiresorptive, anti-inflammatory and osteoinductive drugs, providing the output of functional substances at the implantation site (Figure 5).

### 3.5. Calcium Phosphate Cements for Bone Treatment

Bone tissue is able to spontaneously heal fractures or defects, but regeneration is limited to small areas of the defect. The critical bone tissue defect size is commonly taken to mean the smallest bone defect in a particular bone of a particular living organism that does not spontaneously heal up, or shows less than 10% bone regeneration over its life [102]. Bone grafts are needed to facilitate the repair process if the size of the defect is too large for the bone’s natural ability to heal. Calcium phosphate cements have a similar bone mineral chemistry, and can adapt perfectly to the shape of the defect, making them a convenient option for use as a synthetic bone graft.

Bone is the main storage site for calcium and other ions in the body. Bone fractures, especially during surgical treatment, are associated with changes in microelement homeostasis [103]. Calcium introduction is effective in reducing the risk of postoperative hypocalcemia [104,105,106]. Together with other ions such as magnesium, they have a positive effect on the healing of bone fractures [107]. Ca^2+^ is an important homing signal; it brings together various cell types required to initiate bone remodeling. A high concentration of Ca^2+^ can induce osteoblast proliferation and chemotaxis by binding to an extracellular calcium-sensitive G-protein-coupled receptor [108,109].

Cytokines C-reactive protein (CRP), interleukins IL-1β, IL-6 and tumor necrosis factor alpha TNF-α play a pro-inflammatory role, and are important mediators of the immune response and inflammatory response. The disordered expression of IL-1β, IL-6 and TNF-α is considered an effective biomarker of inflammation associated with the development and process of fractures [110]. An increase in the content of Ca and Mg, and a decrease in inflammatory cytokines, are observed after short-term treatment with trace elements. Their addition can be an effective way to combat inflammation, as it contributes to the restoration of bone fractures [105]. The disordered expression of IL-1β, IL-6 and TNF-α is considered an effective biomarker of inflammation associated with the development and process of fractures [110]. There is an increase in the content of Ca and Mg, and a decrease in inflammatory cytokines, after short-term treatment with trace elements. The addition of trace elements can be an effective way to fighting inflammation, and contribute to the reconstruction of bone fractures [105].

Calcium phosphate cements can be used to strengthen vertebral bodies affected by osteoporosis [111] in cranio-maxillofacial surgery [112,113], as well as for treating fractures and revisions, mainly to fill defects resulting from the surgical removal of cysts and tumors, trauma and osteolytic defects, or in the surgical treatment of infections [114].

**Figure 6:**
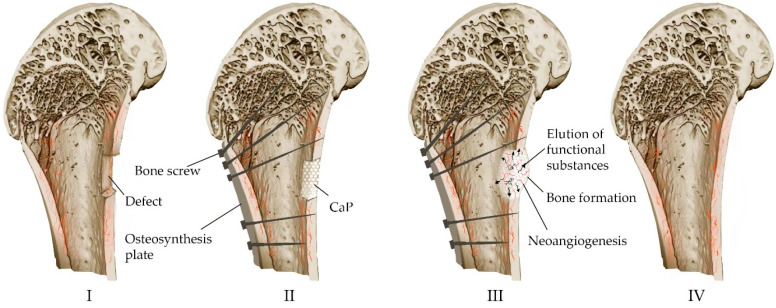
Scheme of replacement of bone defects with the help of calcium phosphate material: (**I**) Formation of a defect along the boundaries of viable tissues (resection of compromised areas as a result of high-energy trauma, inflamed areas in osteomyelitis, tumor lesions, etc.). (**II**) Installation of metal structures for mechanical strength for the period of healing and implantation of osteoplastic material to close the defect. (**III**) Reconstruction of osteoplastic material in the defect, resorption, biodegradation and formation of bone. (**IV**) Bone remodeling.

Resorbable calcium phosphate cements prepared by low-temperature technology containing drugs or other biologically active substances and cells can potentially act as multifunctional carriers and drug delivery systems [115,116]. Figure 6 shows the process of bone defect replacement during the implantation of calcium phosphate material in the form of a block, granules or cement paste.

Currently, calcium phosphate materials are used in the field of traumatology and orthopedics in the form of powders, granules, blocks, cements and coatings on metal implants [117,118,119,120,121,122]. Due to the high strength of ionic bonds, calcium phosphate materials are brittle and cannot carry heavy loads. They are used as osteoplastic materials in small defects or operated with the additional use of structures on which the load is shifted.

Calcium phosphate cements are osteoconductive and bioactive, and they integrate into bone tissues without forming a connective tissue capsule [123]. The cements are resorbable, moldable and easy to handle. They can be injected into bone cavities under conditions of limited surgical access, and completely fill cracks (defects) in situ in the operating room, thus minimizing surgical intervention. They provide good fixation and close contact between the bone and the material [124]. Cements harden, acquiring their own mechanical strength [121].

## 4. Concepts for the Production of Calcium Phosphate Cements

Calcium phosphate blocks and granules obtained as a result of the hardening of calcium phosphate cement are a promising type of scaffold for restoring bone defects due to their similarity in composition with the mineral component of bone tissue, high specific surface area, and increased resorption rate compared to matrices obtained by high-temperature processing. In addition, the volumetric incorporation of functional substances at the stage of mixing the components can be used to change the release kinetics, in contrast to the surface impregnation of high-temperature calcium phosphate materials [117,125,126,127,128]. Matrixes can be formed by casting cement paste into molds, by interacting pre-pressed initial components in an aqueous medium via setting and hardening, and by 3D printing; they can serve as a substrate in tissue engineering and are an excellent platform for incorporating functional substances [117,129,130,131].

Cements are usually obtained from powders of one or more calcium phosphates and an aqueous solution. When mixing powders of calcium phosphates and an aqueous solution, a paste is obtained. It hardens within minutes. Another form of supply of calcium phosphate cement is pre-mixed cement components in the form of a cement paste. There are three main approaches used to prepare premixed cements: (1) one-component cement in the form of calcium phosphate paste mixed with a non-aqueous liquid (hardening when mixed with biological fluids); (2) two- or more-component cement in the form of pastes (hardening when mixed); (3) one-component cement in the form of a paste of calcium phosphate in an aqueous liquid, followed by freezing (hardening during thawing). This form of delivery is presented in the commercial products of VitalOs from CalciphOs/Produits Dentaires SA and VELOX from InnoTERE GmbH [132].

The constant solidification volume of cements and low heat release (minor exothermicity at low heat release rate) [88,125,133] are properties that that enable their use.

An increase in mechanical strength is facilitated by the isolation of particles of non-equiaxed morphology (lamellar, needle-shaped), which provide mechanical engagement. The cements fuse well with the bone, gradually dissolving and being replaced by new bone tissue.

Powders and blocks of cements can be sterilized by γ-irradiation without the loss of biocompatibility and bioactivity [134]. Steam sterilization, ethanol sterilization, and ethylene oxide sterilization with complete degassing after sterilization are also mentioned [128,131,135]. Gamma radiation for powder components and filtration for liquid components are used in the sterilization of cement formulations [136].

Calcium phosphate cements can be divided into two categories according to the final product, despite the large number of possible preparation methods: (1) based on hydroxyapatite; (2) based on dicalcium phosphate dihydrate CaHPO_4_·2H_2_O (DCPD) (or brushite) [121,125,137,138].

A significant part of the commercial cement materials used in medicine for the treatment of bone tissue defects contain minerals belonging to the CaO–P_2_O_5_–H_2_O system [139]. The production of calcium phosphate cements is usually realized according to two scenarios, with two chemical processes: acid–base interaction and hydrolysis.

As a result of the acid–base reaction, cements based on hydroxyapatite Ca_10_(PO_4_)_6_(OH)_2_ (HA) (Ca/P = 1.67)/calcium-deficient hydroxyapatite Ca_10−x_(HPO_4_)_y_(PO_4_)_1−y_)_6_(OH)_2_ (CDHA) (Ca/P = 1.5) and brushite CaHPO_4_·2H_2_O (DCPD) (Ca/P = 1)/monetite (dicalcium phosphate anhydrite) CaHPO_4_ (DCPA) (Ca/P = 1) are obtained by reacting tetracalcium phosphate Ca_4_(PO_4_)_2_O (TTCP) (Ca/P = 2) or β-tricalcium phosphate β-Ca_3_(PO_4_)_2_ (β-TCP) (Ca/P = 1.5) with DCPD (Ca/P = 1)/DCPA or monocalcium phosphate monohydrate Ca(H_2_PO_4_)_2_·H_2_O (MCPM) (Ca/P = 0.5), without the formation of acidic or basic co-products. Monetite is formed under conditions of water deficiency and low pH [120,140].

Only one precursor is involved in the hydrolysis reaction. When mixed with the liquid phase, it becomes hydrated. The reaction proceeds with the dissolution of PO4^3−^ and Ca^2+^ ions. On the surface of the α-tricalcium phosphate α-Ca_3_(PO_4_)_2_ (α-TCP) or amorphous calcium phosphate Ca_3_(PO_4_)_2_ (ACP), particles of precipitated crystals of calcium-deficient hydroxyapatite form during the hydrolysis process.

Examples of possible combinations of the main initial components used in the production of calcium phosphate cements as commercial products are shown in Table 1.

HA and brushite cements differ significantly in setting time, mechanical strength, resorption rate in the body, and pH values. In this regard, approaches to improving the properties of cements differ.

HA cements are long-hardening; the setting time is no earlier than 30 min. A decrease in cohesion upon contact with blood and partial mixing with it may occur during prolonged hardening, and this will lead to a loss of quality or migration from the implantation site [141,142,143].

Miyamoto and colleagues stated that the cement sets consistently despite partial decomposition of the cement paste on contact with blood during setting. However, fast-setting cements may be less sensitive to contact with blood [144].

Particle size and the degree of crystallinity strongly affect the degree of reactivity of cements and, as a result, the rate and integral completeness of the reaction [145,146]. ACP is the most reactive because it has the least stable crystalline phase. It is followed by α-TCP and finally β-TCP [147]. A decrease in the particle size leads to an increase in the surface area, and an increase in the reactivity and the reaction rate [117,148,149].

The introduction of a seed of crystallization increases the rate of hydration and hardening [149,150,151]. The seed plays the role of a substrate that can be used for heterogeneous nucleation. The nucleation barrier does not exist if the substrate is identical to the nascent crystal.

HA cements are relatively insoluble in aqueous solutions at neutral pH. Solubility increases at acidic pH values, as their own pH values correspond to physiological values.

Brushite cements are fast-setting, with setting times less than 1 min, as well as being soluble, highly bioresorbable and biocompatible, and relatively acidic (pH around 4).

The rate of resorption of cements in the body can be regulated by various technological parameters: L/P ratio, porosity, phase composition (ion substitutions, introduction of additional components of cement powder), crystallinity, as well as the presence and quantity of additives.

The introduction of additives into the system or due to ionic substitutions can increase the setting time of cement. For example, in the production of brushite cement, the setting time and compressive strength were increased by replacing calcium in TCP with magnesium by up to 10%. Moreover, the initial setting time increased to 33 min with magnesium content [152]. Changing the particle size of the initial components affects the setting time and strength of the cement.

In the literature, the compressive strength of HA cements is 20–83 MPa, while that of brushite cements −1–24 MPa, and tensile strength of brushite cements −0.7–4.5 MPa, and that of HA cements is up to 15 MPa. Such data were obtained by measuring the strength under different conditions. Cement samples dried in air or at a slight increase in temperature have the maximum strength values.

Among the ways to increase the strength of cements are the following: reducing the liquid/powder ratio (L/P or LPR) when receiving cement paste, introducing additives, changing the particle size [153,154], and introducing fillers (fibers, granules) inert with respect to the cement stone. As fillers, granules or fibers of the inorganic compounds β-TCP, HA, gypsum, bioglass, carbon, silica, wollastonite and zirconium dioxide can be used. They are biocompatible, non-resorbable or poorly resorbable compounds [155,156,157], with the exception of resorbable β-TCP and some bioglasses.

Fibers and granules made from resorbable polymers such as polylactic acid (PLA) [158], polylactide-co-glycolide (PLGA) [159], polyvinyl alcohol (PVA) [160], gelatin or chitosan [161,162] increase initial strength and create porosity over time. In this regard, the characteristics of the cement stone change; this affects the behavior of the material in vivo. Such fillers can be attributed to additives as modifiers of cements.

Calcium phosphate cements have a porosity of 30–60% by volume, typically depending on the composition of the cement. The porosity is mostly open. Pore sizes up to 1 micron do not provide bone tissue ingrowth, and resorption occurs from the surface of the material. The porosity of calcium phosphate cements is due to excess mixing water. As the amount of water decreases, the porosity decreases, and hence the mechanical properties improve. This leads to a decrease in the resorption rate and the deterioration of rheological properties [125].

The absence of macroporosity is attributed to the disadvantages of calcium phosphate cements, in particular HA, while the presence of microporosity is an advantage. This is due to the fact that microporosity creates a surface microrelief for cell retention, and improves osteogenesis [162,163,164,165,166]. In addition, microporosity is a positive factor in calcium phosphate cements–functional substance delivery systems. However, microporosity reduction due to prepressing is used to obtain cement scaffolds with increased strength [167], and in combination with removable fillers to form macroporosity, it reduces strength to a lesser extent [126,168]. Another approach to reducing porosity is to reduce the particle size of the original components [169].

Ionic substitutions can also affect porosity. For example, when using silicon beta-tricalcium phosphate (Si-β-TCP) in the preparation of brushite cement, the pore size decreases in direct proportion to the amount of Si from micro- to nano-size, and the surface area increases [170].

Macroporosity provides blood with access to contact surfaces, and allows angiogenesis [162,171]. The absence of macroporosity is solved by introducing soluble additives and removing them before or after implantation (in vivo).

### 4.1. Osteogenic Ionic Substitutions in Calcium Phosphate Cement

The calcium of hydroxyapatite and brushite can be replaced by the following cations: magnesium, radium, strontium, barium, sodium, zinc, etc. The phosphate ions of hydroxyapatite can be replaced by carbonate or silicate ions. Currently, more than 30 osteotropic microelements (copper, strontium, zinc, barium, aluminum, silicon, fluorine) are known, the lack or excess of which can cause various rickets, leading to skeletal growth arrest and other consequences. Substitutions with magnesium ions, strontium and carbonate ions are already used in the preparation of commercial products (see Table 1). For example, calcium carbonate is used to produce carbonate hydroxyapatite as the target phase in commercial products Calcibon^®^ and Norian. Calcium carbonate participates in the hydrolysis process together with α-TCP, facilitating the incorporation of CO_3_^2−^ ions into the crystal lattice [172]. A negative charge deficiency is formed when PO_4_^3−^ is replaced by CO_3_^2−^ or HPO_4_^2−^; it is compensated by calcium deficiency and the replacement of Ca^2+^ ions by Na^+^.

It is known that the presence of the bioactive Mg^2+^ ion in the structure of calcium phosphates plays a significant role in the biological process and stimulates bone formation [173,174,175,176,177]. Magnesium is an obligate cofactor of alkaline phosphatase and many enzymatic reactions, inhibits the formation of osteoclasts, and participates in the synthesis of bone collagen, cell proliferation and differentiation, and the interaction of the cell with the matrix, as well as the normal functioning of organs [178,179,180,181,182]. Mg^2+^ ions in HA cement can reduce the setting time, and increase it in brushite cement. This is a beneficial effect for both cements [152,177].

One of the important features of the substitution of calcium ions Ca^2+^ for magnesium ions Mg^2+^ in brushite is the stabilization of the phase composition [180,183,184,185]. Magnesium ions suppress the formation of less soluble calcium phosphates (hydroxypatite, tricalcium phosphate, octacalcium phosphate) in vivo in brushite materials [185,186,187,188,189]. Magnesium acts as a strong inhibitor of the crystal growth of less soluble calcium phosphates. Magnesium–calcium phosphate cement does not recrystallize with time, in contrast to brushite, and retains a high resorption rate [181,190].

Strontium is very close to calcium in nature, and therefore they are associated in the metabolic processes of bone tissue. Strontium accumulates mainly in the newly formed bone and in the areas of ossification, being part of the trabeculae. Ca^2+^ and Sr^2+^ ions can be replaced by Sr^2+^ ions in the cement composition. Sr^2+^ has been proven to be effective in stimulating osteogenesis, and in the treatment of osteoporotic bone fractures. It acts as an inhibitor of bone resorption and as a stimulator of bone formation [177,191].

The replacement of calcium ions with zinc ions significantly contributes to the formation of new bone without an inflammatory reaction. With the introduction of zinc into the composition of the cement, the proliferation of primary human mesenchymal stem cells is significantly stimulated, and the activity of alkaline phosphatase increases [192,193,194,195].

The co-introduction of Si and Zn ions increases the rate of resorption of calcium phosphate cement significantly, as well as angiogenesis and osteogenesis, due to the synergistic effects of Si and Zn on biostimulation and immunoregulation [196].

There are two approaches to ionic substitution: introducing additional compounds into the composition of the powder part of cement [195,196], or by doping one of the original components [120,170,194,197].

In addition to alkaline earth metals, ions of other bioactive metals such as Cu^2+^, Co^2+^, Cr^2+^ and Ga^3+^ at low doses can also accelerate bone tissue repair [88]. Cu^2+^-doped cement has antibacterial properties and stimulates angiogenesis, bone marrow mesenchymal stem cell (BMSC) differentiation and bone mineralization. Co^2+^ shows conflicting results. Cr^3+^ has a positive effect on bone formation, and supports the proliferation of osteogenesis precursor cells and osteoclast resorption. Ga^3+^ has a positive effect on the synthesis of mature organized collagen and an inhibitory effect on osteoclasts [88]. The inhibitory effect of Ag-doped calcium phosphate cements on pathogenic Escherichia coli has been proven [197].

Ionic substitutions are used to improve the properties of cements for the treatment of bone cancer. These ions include holmium (Ho) and samarium (Sm) ions, and a collection of manganese (Mn), lanthanum (La), strontium (Sr), cobalt (Co), and iron (Fe) ions, including iron oxide and magnetite [198].

The medical impact of any micronutrient needs to be assessed in order to establish toxic thresholds. For example, the use of high doses of strontium causes defects in bone mineralization [199], while high doses of zinc cause a slowdown in bone repair [200] and Cu^2+^ cytotoxicity [88].

### 4.2. Influence of Modifier Additives on the Properties of Cements

Inorganic and polymer additives greatly affect the properties of cements. They are necessary to obtain medical devices suitable for use in the field of traumatology and orthopedics (Table 2).

### 4.3. Porosity and Features of the Pores

Slowly resorbed HA cements require the formation of macroporosity. It increases the surface area and the rate of resorption. The pores provide fluid flow (perfusion in the case of interconnected porosity), as well as the migration and proliferation of osteoblasts in cements, and vascularization. In addition, in the presence of pores, the stability of the tissue–implant interface improves. This is due to a larger surface area for cell proliferation and the regeneration of new tissue [121].

Depending on the size of the pores in calcium phosphate cement, they are classified into micropores (pore diameter <1 microns), mesopores (pore diameter 1–100 microns) and macropores (pore diameter >100 microns) [121]. The size of osteoblasts is about 10–50 microns [212]. However, osteoblasts prefer larger pores (100–200 microns) for the regeneration of mineralized bone after implantation. Macrophages can enter these pores, destroy bacteria and cause the infiltration of other cells involved in colonization, migration and vascularization in vivo [213]. As a rule, a pore size of ≥300 microns is required for the formation of new bone and vascularization. The minimum allowable size is ≈100 microns [121,214,215]. The pore size <100 microns prevents angiogenesis [216]. According to the results of many studies, bone tissue is formed in such pores [121,215,217]. Small pores favor hypoxic conditions and induce osteochondral formation before osteogenesis. Large pores with good vascularization can lead to direct osteogenesis (without prior cartilage formation) [215].

Pores larger than 300 microns are well supplied with oxygen and nutrients. This promotes vascularization in new bone tissue and, accordingly, osteogenesis. [215,216,218,219,220].

Another important parameter of the porosity of calcium phosphate cements is the interconnectedness of the pores. Porosity can be open and closed. Open porosity has an advantage. The interconnected open microporous system ensures good impregnation of the material with biological fluids and oxygen diffusion, and creates a surface roughness. Roughness plays an important role in the adsorption and retention of osteogenic cells on the implant surface. Macropores facilitate cell infiltration and migration into the scaffold, as well as angiogenesis and osseointegration [221,222,223,224]. Macroporosity allows cells to migrate and proliferate into the matrix, provides easy access for cells (inflammatory, stem) and soluble proteins, including signaling molecules and osteogenic growth factors, and enhances active (cell-mediated) and passive (solubility) cement resorption. [224].

Interconnected macroporosity is a necessary but not sufficient requirement. The shape and architecture of pores are of great importance to the behavior of cement scaffolds in vivo. The spherical concave surface of the pores of various types of materials has a positive effect on in vivo behavior [224,225,226]. Triangular, rectangular and elliptical pores support angiogenesis and faster cell migration due to their greater curvature [218].

The closed spaces of spherical macropores act as niches for the differentiation of mesenchymal stem cells into osteoblasts due to the presence of calcium and phosphate near the scaffold, and osteoinductive growth factors. Such a microenvironment does not occur in an open structure where ions and proteins diffuse easily [224].

Thus, scaffolds based on calcium-deficient hydroxyapatite with spherical, concave macropores form due to the release of CO_2_ in the process of cement stone formation, and these cause significantly more intense ectopic bone formation during intramuscular implantation than 3D-printed scaffolds with convex, prismatic macropores. Ectopic osteogenesis in 3D-printed scaffolds was present only at the corners where a concave surface formed. The rate of local growth of tissue in concave spaces is proportional to the curvature of the concavity. Moreover, no difference in angiogenesis was observed with different pore shapes [224].

### 4.4. Resorbability

The main difference between the end products of hydroxyapatite and brushite calcium phosphate cements is their solubility and resorption rate. Brushite cements are more soluble than HA. Therefore, they are more rapidly resorbed in vivo [125,227]. Ideally, the rate of biodegradation of calcium phosphate cements should be nearly the same as the rate of new bone formation. This will ensure the gradual restoration of the mechanical properties of the new bone tissue. Although HA cement resorbs faster than high-temperature HA, resorption can take several years to decades [123].

The resorption of both types of cements proceeds from the periphery to the center (creeping substitution) at the bone–cement boundary [123]. Osteoclast-like cells are present on the interface. They resorb cement stone. At the sites of resorption, new bone tissue is formed, and the integrity between the bone bed and cement is preserved (osteotransductive property of cement) [228].

In vivo resorption is carried out in two different ways: (i) passive resorption by dissolving cement stone in extracellular fluid and (ii) active resorption due to cell activity [121].

The rate of passive resorption by extracellular fluid depends on the properties of cements—L/P ratio, porosity, surface area, phase composition, Ca/P ratio and crystallinity—as well as the microenvironment properties—pH and perfusion by body fluids [229,230].

The active resorption of calcium phosphate cements is mediated by giant cells and osteoclasts. Macrophages absorb fragmented cement particles [231,232]. Macrophages are among the first cells to enter the fracture site. They contribute to the initial inflammation and rehabilitation of the injury site, as it was thought for a long time. [2]. The main role of macrophages is to regulate bone regeneration during normal homeostasis and during fracture healing. In addition to macrophages, osteoclasts play complex roles in bone growth and regeneration [233,234]. The mature osteoclast is tightly attached to the mineral surface. It lowers the pH locally near the biomaterial and dissolves the inorganic calcium phosphate underneath [235].

The rate of resorption of calcium phosphate cements affects cell proliferation. This is due to the interaction of the released calcium with the extracellular calcium-sensitive receptor associated with the G-protein [108,109]. In this regard, cellular proliferation is higher in carbonate-substituted HA cements [126] than in HA cements.

Brushite cements show a higher rate of resorption than HA cements, but there is a possibility of the recrystallization of brushite into low-soluble phases [187,236]. The resorption rate of brushite cement should be increased or decreased depending on the purposes of its use (bone restoration, drug delivery system). To prevent the recrystallization of brushite into low-resorbed phases (for example, hydroxyapatite), a highly resorbed phase of newberyite is introduced into the composition [76,120]. Slowing down the rate of resorption can be achieved by inhibiting osteoclast-mediated resorption, for example, by including simvastatin to stimulate bone formation and inhibit cement resorption [232].

A chemical approach to improve cell adhesion and osteogenic differentiation aims to create functional groups on the surface of matrices, such as −COOH and −NH_2_. Through functional groups, the surface binds to proteins using hydrogen bonds [237].

An increase in the resorption rate of HA cement is possible due to the presence of macroporosity. The active and passive resorption of calcium phosphate cements is enhanced in the presence of macroporosity. It allows cells to migrate and proliferate into the matrix, and increases the surface area [116]. The introduction of particles of poly(D,L-lactic-co-glycolic acid) (PLGA) increases the rate of dissolution of the cement matrix due to the formation of marcoporosity and the presence of acidic monomers (lactic and glycolic acids). Acidic monomers accelerate matrix degradation [208].

The structure of macropores affects not only bone formation, but also the resorption of the material. Matrices with concave macropores have a higher cell-mediated resorption compared to matrices with convex prismatic pores. The interstitial microenvironment in concave pores influences osteoclastic activity [224].

Preclinical studies of calcium phosphate cements unambiguously confirm their biocompatibility, bioactivity and resorbability. However, the resorption rate depends on a large number of the technological parameters of the preparation of cements (precursor synthesis methods—firing temperature, particle size, crystallinity; cement paste and cement stone parameters—he presence and amount of additives, strength, porosity, cohesion, phase and chemical composition, phase distribution, pH) and the different osteogenesis processes in different animals, as well as the place of implantation. The bone metabolism of sheep, pigs (1.2–1.5 mm/day), dogs (1.5–2.0 mm/day) and goats more closely mimics human bone physiology in terms of bone metabolism (1.0–1.5 mm/day), long bone size, and mechanical loading conditions, but there are many other differences. Bone metabolism and regenerative capacity are faster in rodents and rabbits [138].

The difficulty in predicting the clinical outcome of calcium phosphate cement implantation lies in the individual characteristics of the patients, such as age, gender, metabolism and comorbidities (e.g., osteoporosis or inflammatory diseases). However, non-clinical evaluation is important for the study of material behavior, including comparative evaluations with existing commercial medical devices.

## 5. Calcium Phosphate Cements as Carriers of Functional Substances

The incorporation of active molecules into calcium phosphate cement can be achieved by dissolving it in the liquid phase, by mixing with the powder phase, or by simultaneously mixing with the powder and liquid phases [238], including with granules of resorbable fillers. The surface adsorption of drugs to the cement surface by incubating the scaffold in a drug solution is another possible approach. Surface impregnation for cements is rarely used. The kinetic release of drugs depends on the functionalization, microstructure and resorbability of the CPC matrix [116,129]. The gradual release of drugs is ensured by a homogeneous distribution in the cement stone volume, which can be effective in the treatment of various bone diseases such as tumors, osteoporosis or osteomyelitis [115]. Cells, injectable calcium phosphate cements, and functional agents, used alone or in combination, can promote tissue regeneration in a minimally invasive manner to restore function, reduce risk, reduce complications, and reduce treatment costs [34].

The functionalization of calcium phosphate cements is of great clinical interest for the treatment or prevention of various bone diseases. The main requirement of this for all carrier materials is prolonged elution. The release of functional substances depends on many factors:

- Resorption rate (depends on crystallinity, porosity, phase composition, presence of additives, surface roughness, L/P ratio, molding method, curing conditions, geometric shape and matrix size);
- Size and size distribution of pores (depends on the phase composition, presence and concentration of additives and their nature, L/P ratio, molding method, hardening conditions);
- pH of the cement stone (depends on the phase composition, solubility);
- Solubility of a functional substance (depending on the type, chemical nature);
- The possibility of interaction between the functional substance and the matrix (depends on the chemical formula);
- The size of the molecule of the functional substance;
- Quantity and uniformity of distribution of the immobilized functional substance;
- Method of immobilization of the functional substance;
- Type of supply of calcium phosphate cement (paste or cement stone).

The release of functional substances also depends on environmental factors. There are specific interactions between the ions formed during the dissolution of the drug and the ions of saline buffer solutions. This may play an important role in determining release mechanisms and in shaping the release profile [239]. Saline buffer solutions such as PBS or SBF promote the precipitation of HA and its deposition on the surface of the cement. It acts as a barrier to the diffusion of drugs from the volume, thereby reducing the release rate [137].

Environmental conditions are different in experiments in vivo and in vitro. This affects the release. The amount of vancomycin released in vivo has been reported to be half that of vancomycin released in vitro [240]. It is necessary to take into account that high concentrations of the antibiotic can adversely affect osteogenesis. It was reported that high concentrations of gentamicin sulfate inhibited the production of alkaline phosphatase by cells [241].

The kinetics of release of functional substances from calcium phosphate cements are controlled by diffusion, since matrices are resorbed more slowly compared to the release of functional substances. The mechanism of material resorption can be connected to the diffusion mechanism in the case of a more highly resorbable brushite cement [242,243].

Mathematical models taking into account various internal and external parameters are used to determine the kinetics of drug release from delivery systems (Table 3) [244].

The release curves of functional substances from calcium phosphate matrices most often show bimodal release. In this case, a typical initial release occurs within the first 24 h, followed by a sustained slow release [152]. This corresponds to the Higuchi model. It describes the release of functional substances from matrix systems as a diffusion process, based on Fick’s law and depending on the square root of time. The burst of release in the initial period of time reflects the slight diffusion of functional substances from the surface layer. After this period of time, diffusion from the inner surface of the matrix is somewhat difficult; this slows down the release of functional substances and thereby prolongs the release.

Multi-stage release profiles potentially offer much greater advantages over monotonic drug elution kinetics. The rapid initial release is able to effectively stop the pathological process, while its longer second release phase will gradually support the healing process [245].

The rate of release of functional substances depends on the morphology of the cement stone particles, as it leads to a different degree of adsorption of the substance on the inner surface. Desorption of functional substances from the surface of calcium phosphates with needle morphology of crystals is higher compared to desorption with lamellar morphology [246].

The surface charge of molecules of functional substances increases adsorption with the surface of calcium phosphate cement due to electrostatic attraction. Positively charged functional substances bind to the surface of calcium phosphate, since there are many negatively charged phosphate and hydroxide ions on the surface. Negatively charged functional substances bind to the surface of calcium phosphate with a large amount of calcium ions [247], such as cefaclor and ciprofloxacin, which have carboxyl groups [248]. The functional groups of BMP-2 (hydroxyl, amine and carboxyl) have a high affinity for calcium phosphates.

However, the presence of cephalexin has been reported to inhibit the growth of hydroxyapatite crystals. This is due to the ability of carboxylic acid molecules to be adsorbed on the surfaces of the initial components and nascent crystals, and to inhibit the growth of the target phase [249].

The values of the surface zeta potential are negative for calcium phosphates, but they differ depending on the arrangement of atoms on two types of crystal planes along the (a) axis and along the (c) axis [247]. Plane (a) is rich in positively charged calcium ions, and plane (c) is rich in negatively charged phosphate and hydroxide ions [250]. The different habitus of calcium phosphate crystals determines the different surface zeta potential due to the different arrangement of atoms. Negatively charged functional substances are easily adsorbed on needle-shaped crystals with developed (a) planes. Positively charged functional substances are adsorbed on lamellar crystals with developed (c) planes [246]. Neutral functional substances are adsorbed to a lesser extent and more easily desorbed from the surface of calcium phosphates, for example, metronidazole [248] or di(ethylenediamineplatinum)medronate [246]. Irregular crystals have intermediate zeta potentials and differently oriented planes [247].

Low crystallinity and high specific surface area allow the mobilization of more functional substances [251].

An increase in open porosity leads to an increase in surface area and the faster elution of functional substances from the cement matrix, since the drug solution is released through the phenomenon of capillary flow [252]. The volumetric flow rate of the eluted substance is proportional to the radius of the capillary. The influence of the pore size is more intense when it is similar to the size of the molecules of functional substances. Molecules of functional substances can freely diffuse from much larger pores. Release is controlled by diffusion to a greater extent than by structural factors [253]. This aspect is extremely important in the incorporation of giant molecules of some protein growth factors, such as the bone morphogenetic protein (BMP).

The release rate of functional substances from injection formulations is higher due to the setting period and the initial hardening time until the structure of the cement stone is formed. The kinetics depend on setting time, cohesion, microenvironment and hardening conditions [254,255]. Thus, drug release rates from brushite cement after 3 min and 1 h of curing showed explosive release during the first 8 h and slower release over 4 days. At the same time, the initial substances were found in the composition after 3 min. Most of the reagents turned into brushite after 1 h. The morphology of brushite crystals changed slightly from 1 to 15 h, which is confirmed by the values of the total porosity and tortuosity coefficient [255].

The functional substance should not impair the physical properties of the cements, and the cement should not change the active principle of the functional substance in delivery systems [238]. Brushite cements have a high ion concentration and an acid setting reaction—properties that can reduce or inhibit the effects of certain drugs [238].

In particular, the antibiotic groups of tetracyclines tend to chelate Ca^2+^ ions. This affects the primary formation of the nuclei of crystallization in brushite cements, prevents the deposition of minerals, and inhibits mineralization. Consequently, the incorporation of tetracyclines increases the setting time [256,257].

On the other hand, functional substances loaded in the form of salts into cement stone can affect the structure of the cement stone. For example, the addition of lidocaine hydrochloride increases the size of needle and plate CDHA crystals in TCP-based cement stone [239]. Increasing the size of the crystals ensures a good interaction between the preparation and the surface of the cement crystals. Large needles and plates provide greater adsorption, greater chemical binding and greater dissolution of lidocaine hydrochloride from the surface of cement crystals. This increases the release rate without changing the phase composition of the cement [239,258].

The excipients in the composition of pharmaceuticals ensure their shape, consistency, strength and degradation properties, and can affect the properties of cement stone. For example, sodium stearate inhibits the hydration of α-TCP and slows down the setting reaction. This contributes to an increase in the release rate of the functional substance at the first stage [137].

One of the options for changing the kinetics of the output of a functional agent is its encapsulation in a biodegradable polymer. As a result of encapsulation, the kinetics of elution are limited by diffusion from the polymer. Reduced initial explosive elution of the drug and more prolonged elution at a later date were observed when the substance was incorporated into PGLA microspheres, compared with direct incorporation [259].

The kinetics of elution of functional substances can be determined using the following methods: ultraviolet–visible spectroscopy (UV-Vis), high-performance liquid chromatography (HPLC) and fluorescent polarization immunoassay (FPIA). The electrochemical impedance spectroscopy (EIS) of Pasqual Group positions is a method for determining the amount of a drug without aliquot selection, as required by traditional methods [239].

A brief review of the scientific literature over the past 5 years on calcium phosphate cements as carriers of functional substances for the treatment of bone tissue is presented in Table 4.

## 6. Conclusions

Bone tissue repair is often limited by large defects and associated complications (osteoporosis, infection, and metastasis). Effective treatment strategies are aimed at the resection of the affected area of bone tissue, followed by treatment. Due to the limited availability of the affected bone for systemic therapy and for the prevention of postoperative complications, a local therapy method is used with the delivery of functional substances during surgery, or using minimally invasive procedures. Local prolonged drug delivery systems in effective therapeutic doses to the site of bone disease can contribute to the effective treatment of various bone diseases with the least side effects.

Calcium phosphate cements have a chemical composition similar to the inorganic component of bone tissue. They are biocompatible, resorbable, injectable and self-hardening. They can take the form of a bone defect and fit tightly to the bone bed. The Ca^2+^ ion is an important homing signal; it brings together various cell types necessary to initiate bone remodeling. Ca^2+^ ions affect protein growth factors, and additionally stimulate the expression of osteogenic marker genes.

At present, the most common approach to using calcium phosphate cements as scaffolds is the bulk incorporation of anti-inflammatory, antitumor, antiresorptive, and osteogenic functional substances.

The functionalization of calcium phosphate cements for a specific task requires an understanding of cement systems. Cements are complex systems. Changing one of the many parameters of the system changes the final characteristics of the matrix in a wide range, including the kinetics of elution of functional substances.

In this paper, the diseases of bone tissue are described, in the treatment of which calcium phosphate cements can be used as carriers of functional substances. Knowledge of the pathogenesis of diseases and the methods of treatment used—in particular, drug therapy—can help develop the concepts of directed functionalization for various clinical cases. We tried to isolate and describe in detail the factors that affect the release of functional substances from calcium phosphate cements, as related to the parameters of the cement system and the nature and affinity of functional substances for restoring bone tissue with the matrix.

Over the past two years, interesting reviews have been published by various scientific groups, which indicate an undying interest in calcium phosphate cements. A more detailed description of the properties of injectable calcium phosphate cements and the possibilities of their regulation can be found in the Vezenkova review, where cements are considered from the point of view of obtaining an osteoinductive product [279]. The mechanism of decomposition, recrystallization, resorption assessment and clinical characteristics of biodegradable cement are widely described in the Liu review [280]. A detailed review of composite materials based on cements with bioactive glass was carried out by the Demir-Oğuz scientific group [281].

The functionalization of calcium phosphate cement is one of the most important directions for solving the problems of the treatment and restoration of bone tissue.

The use of calcium phosphate cements as carriers of anticancer drugs and bisphosphonates is addressed in the reviews of Zogakis et al. [282] and Ntep et al. [283]. The possibility of using SaR-bisphosphonate and SaR-doxycycline cements in the non-surgical treatment of osteolytic tumors of the jaws is considered in the review by Ntep et al. Factors for achieving the sustained release of anti-bone cancer drugs are discussed in a review by Pylostomou et al. The influences of drugs, nanoparticles and ion substitutions on the properties of calcium phosphate cements have been analyzed [198]. General issues of functionalization are very thoroughly discussed in the Fosca review article [137].

Based on an understanding of the importance of calcium phosphate cements in the process of bone tissue restoration, and the possibility of functionalization by incorporating various drugs and growth factors directly into the volume of the cement matrix, or via encapsulation in polymer carriers, we assume that future research will aim at solving the problems of interaction between the carrier and functional substances, stabilizing (controlling) the release of functional substances from the matrix, and creating ideal frameworks (structure, geometry, resorption rate) aimed at faster restoration of the integrity of bone tissue.

## Figures and Tables

**Table 1:** The main components of commercial compositions of calcium phosphate cements.

Combinations of Main Components	Product	Commercial Name of the Product
TTCP, DCPD	HA	HydroSet^TM^, Bonesource, Rebone
TTCP, DCPA	HA	Cerapaste, Rebone Gutai, DirectInject
α-TCP, DCPA, CaCO_3_, HA	HA	Calcibon^®^
α-TCP	HA	SKaffold^TM^
α-TCP, CaCO_3_, MCPM	HA	Norian ^®^SRS, Norian ^®^CRS
α-TCP, TTCP, DCPD, HA, Mg_3_(PO_4_)_2_	HA	Biopex^®^-R
α-TCP, Mg_3_(PO_4_)_2_, MgHPO_4_, SrCO_3_	HA	KyphOs^TM^
β-TCP, DCPD	Brushite	JectOS^®^
β-TCP, DCPD, MgHPO_4_∙3H_2_O	Brushite	ChronOS™ Inject
β-TCP, DCPD, MCPM, CaSO_4_∙2H_2_O, H_3_PO_4_	Brushite	VitalOs
β-TCP, H_3_PO_4_	Brushite	Eurobone^®^

Additional components (polymers, setting regulators) are not listed in the table.

**Table 2:** Additives and their effect on cement.

Additive	Form of Submission	Type of Cement	Effect on Cement	Reference
Glycolic acid	Water solution	Brushite	Setting retardation	[201]
Na_x_H_3−x_PO_4_ (Na_2_HPO_4_, NaH_2_PO_4_)	Water solution	HA	Increased strength, setting acceleration	[144] [149]
NaHCO_3_	Particles	HA	Macroporosity	[171]
Na_2_HPO_4_	Particles	HA	Macroporosity	[171]
Pyrophosphate ions (P_2_O_7_^4−^)	Water solution	Brushite	Setting retardation	[153] [125] [154] [202]
Sulfate ions (SO_4_^2−^)	Water solution	Brushite	Setting retardation	[153] [125] [154] [202]
Citrate ions (C_6_H_5_O_7_^3−^)	Water solution	Brushite	Setting retardation, reduced pH, increased strength	[201] [153] [125] [202] [167]
Sodium chloride	Particles	HA	Macroporosity	[126]
Lactic acid	Water solution	HA	Increased injectability, setting acceleration	[203]
Sodium glycerophosphate	Water solution	HA	Increased injectability	[203]
Hyaluronic acid, sodium hyaluronate	Water solution	HA	Increased strength, osteoinduction	[204]
Sodium alginate	Water solution	Brushite	Increased strength, cell proliferation, reduced injectability, pH	[205]
Cellulose esters	Water solution	HA	Increase in cohesion, injectability, decrease in resorption rate	[206]
Polyethylene glycol	Particles	HA	Macroporosity	[117]
Chitosan	Water solution	HA	Increased injectability, setting acceleration, increased strength	[203]
Chitosan	Fiber	HA	Increased strength	[161]
Glycerin	Water solution	HA	Increased injectability, setting retardation	[203]
Collagen	Water solution	Brushite	Increased strength, cohesion, cell adhesion	[207]
Polylactide	Particles/Fiber	HA	Increased strength, porosity	[158]
Polylactoglycolide	Particles/Fiber	Brushite	Increased strength, cell growth retardation	[159]
Polylactoglycolide	Particles/Fiber	HA	Macroporosity, increase in resorption rate	[208] [206]
Sucrose	Particles	HA	Macroporosity	[171] [209]
D-Mannitol	Particles	HA	Macroporosity	[210]
Gelatin	Particles	Brushite	Macroporosity, setting acceleration, reduced strength	[162]
Poly(N-vinylpyrrolidone	Particles	HA	Macroporosity, increase in cohesion, injectability	[163]
Poly(N-vinylpyrrolidone	Water solution	HA	Increase in cohesion	[211]
Polyvinyl alcohol	Particles/Fiber	HA	Increased strength	[160]
Biosurfactants	Water solution	HA	Macroporosity, reduced strength	[135]

**Table 3:** Mathematical models for the determination of the kinetics of drug release.

Model	Mathematics Equation	Applications
Zero order	$Q_{t}=Q_{0}-K_{0}\cdot t$	Can be used to describe the drug dissolution of several types of modified-release pharmaceutical dosage forms, as in the case of some transdermal systems, as well as matrix tablets with low soluble drugs, coated forms, osmotic systems, etc.
First order	$\log Q_{t}=\log Q_{0}+\frac{K_{1}}{2303}$	Can be used to describe the drug’s dissolution in pharmaceutical dosage forms such as those containing water-soluble drugs in porous matrices.
Hixson–Crowell	$M_{0}^{1/3}-M_{t}^{1/3}=K\cdot t$	Can be used for different pharmaceutical dosage forms such as tablets, where the dissolution occurs in planes that are parallel to the drug surface if the tablet dimensions diminish proportionally, in such a way that the initial geometrical form keeps constant all the time.
Higuchi	$f_{1}=Q=A\sqrt{D\left(2C-C_{s}\right)C_{s}t}$ $f_{1}=Q=K_{H}\sqrt{t}$	Can be used to describe the drug dissolution from several types of modified-release pharmaceutical dosage forms, as in the case of some transdermal systems and matrix tablets with water-soluble drugs.
Weibull	$W=1-e^{-\left(\frac{t}{T_{a}}\right)}{}^{b}$	Can be used for comparing the release profiles of matrix-type drug delivery.
Korsemeyer– Peppas	$\frac{M_{t}}{M_{0}}=at^{n}$	Can be used to the linearization of release data from several formulations of microcapsules or microspheres.
Hopfenberg	$\frac{M_{t}}{M_{\infty }}=1-\left[1-\frac{K_{0}t}{C_{0}a_{0}}\right]{\;}^{n}$	Can be used for identification of the mechanism of release from the optimized oily spheres using data derived from the composite profile, which display site-specific biphasic release kinetics.
Baker–Lonsdale	$f_{1}=\frac{3}{2}\left[1-\left(1-\frac{M_{t}}{M_{\infty }}\right){\;}^{2/3}\right]-\frac{M_{t}}{M_{\infty }}=kt$	Can be used in the linearization of release data from several formulations of microcapsules or microspheres.
Gompertz	$X\left(t\right)=X_{max}exp\left[-\alpha e^{\beta \;\log t}\right]$	Can be used for comparing the release profiles of drugs with good solubility and intermediate release rates.

**Table 4:** Calcium phosphate cements as carriers of functional substances for the treatment of bone tissue.

Modifying Agent	Type of Cement	In Vivo/In Vitro	Summary	Year	Reference
Doxycycline hyclate	HA	In vitro	Cement with chitosan solution containing *Doxycycline hyclate* (CPC + DOX) had a strong antibacterial effect, with a 4-log colony-forming unit reduction effect against *S. aureus* and *P. gingivalis*. Alkaline phosphatase activity, mineral synthesis, and osteogenic gene expressions for CPC + DOX 5 mg group were much higher than control group. Alginate microspheres encapsulating stem cells co-cultured with cement protected cells during cement setting. DOX did not compromise the osteogenic induction.	2021	[260]
Vancomycin	HA + calcium sulfate	In vivo	Vancomycin-laden calcium phosphate cement (CPC/V) and Vancomycin-laden polymethylmethacrylate cement (PMMA/V) in the osteomyelitis model were compared. Stronger bone-healing enhancement was shown by the CPC/V group, further proving the advantages of CPC/V over PMMA/V as orthopedic antibiotic carrier.	2019	[261]
Vancomycin	HA	In vivo	Vancomycin-laden calcium phosphate cement (CPC/V) and Vancomycin-laden polymethylmethacrylate cement (PMMA/V) were investigated. CPC/VCM released greater concentrations of VCM for a longer period of time within the 24 weeks than PMMA/VCM. Moreover, CPC/VCM released 1.4 to 26.1-fold more VCM than PMMA/VCM.	2021	[223]
Gentamicin sulphate	HA + bioactive glasses	In vitro	Cement-based matrices with the addition of 10 wt.% bio-glass with gentamicin immobilized on their surfaces were pressed under pressure at 0.7 MPa. The matrix was antibacterial and gentamicin did not significantly delay the setting of cement.	2022	[262]
Gentamicin sulphate	HA	In vitro	Injectable radiopaque cement containing BaSO_4_ and gentamicin sulfate in the form of solid lipid microparticles (range 75–250 µm) obtained by spray-cooling and containing 20% gentamicin sulfate was investigated. Thanks to the use of spray-congealed microparticles, gentamicin sulphate can be added to the cement composition without the lengthening of the setting times and the worsening of the compressive strength observed when the drug is loaded directly into the cement powder without the protection of the microparticles.	2020	[263]
Gentamicin sulphate	HA	In vitro	Cement containing spherical balls of cross-linked gelatin–alginate hydrogels impregnated with gentamicin sulfate has a long-lasting antibacterial effect, good cell adhesion properties and good biocompatibility. Composites have reduced strength, and the HA-phase of the product formed by cement is slightly reduced due to the properties of hydrogel.	2021	[241]
Vancomycin	HA	In vivo (clinical)	Ninety-eight patients with chronic osteomyelitis were randomly allocated into the research group or the control group. Vancomycin-loaded calcium phosphate cement (CPC-V) was used to implant 49 patients in the case of one-stage treatment after debridement. One-stage vancomycin-loaded CPC implantation osteomyelitis lesions fill the die cavity, enable patients to continue to fight infection, induce bone defect osteogenesis, reduce the recurrence of chronic osteomyelitis, and are an effective method for treating chronic osteomyelitis.	2021	[264]
Amikacin + vancomycin	HA	In vitro In vivo	Antibacterial calcium phosphate cement containing phosphoserine and antibacterial drugs, amikacin and vancomycin, demonstrated a continuous release pattern up to 60 and 90 days, respectively. In vivo studies on a sternotomy model in rats infected with S. aureus and E. coli demonstrated significant inhibitory activity compared to the infected control group.	2023	[265]
Vancomycin	HA	In vitro In vivo	Based on pressed carbonate-substituted hydroxyapatite cement containing vancomycin (CPC/V), a method is developed for the preparation of macroporous matrices that combine bioresorbability and osteoconductivity. The effect of CPC/V is demonstrated in the regions by inhibiting the growth of bacterial cultures. As proven in in vivo experiments on the implantation of bones, the matrix is a therapeutically effective carrier of antibacterial substances in the treatment of septic purulent inflammations of bone tissues.	2022	[117]
Ciprofloxacin	HA	In vitro	Biocomposite scaffolds comprising self-assembling peptide hydrogel AcN-RADARADARADARADA-CONH_2_ (RADA), calcium phosphate cement and ciprofloxacin (RADA-CPC-C) prevent bacterial infection and simultaneously enhance osteoblast proliferation, differentiation and mineralization. The amount of ciprofloxacin released from RADA-CPC-C was higher than that of CPC-C in vitro, particularly in the initial 24 h, which may help prevent early infections in the postoperative period.	2021	[266]
Gentamicin sulphate	HA	In vitro	Injectable non-cytotoxic calcium phosphate cement with carboxymethyl cellulose solution containing gentamicin sulfate showed suitable setting and mechanical properties, and injectability around 87%. The antibiotic released after at least 14 days is highly effective against S. epidermidis, but also presents some antibacterial activity against S. aureus.	2022	[267]
Doxycycline hyclate	HA	In vivo	The better performance of doxycycline hyclate-loaded macroporous CPCd in comparison with the doxycycline hyclate-loaded microporous CPCd has been proven via the higher release of doxycycline hyclate, and it promotes bone cell activity, vascularization, and the better distribution of the antibiotic.	2019	[268]
Rifampin Sitafloxacin	Brushite	In vivo	Osteoconductive 3D-printed CaPS with rifampin and sitafloxacin demonstrates more efficacious bacterial colonization outcomes and bone growth in a single-stage revision in comparison to gentamicin-laden PMMA requiring a two-stage revision. Significant increase in bone formation was observed for 3D-printed CaPS incorporated with rifampin at 3 and 10 weeks.	2019	[130]
Gentamicin sulphate	Brushite	In vitro	Calcium phosphate bone cement contained porous granules of β-tricalcium phosphate and hydroxyapatite saturated with gentamicin sulfate, which released an antibiotic at different rates with a concentration greater than the minimum inhibitory concentration of staphylococcus.	2019	[269]
5-fluorouracil	HA	In vitro	Various optimized polymeric solutions (both hydrophilic Soluplus (SOL) and polyethylene glycol (PEG) and a combination of both) containing a model anticancer drug 5-fluorouracil (5-FU) were used to homogenously coat the various 3D-printed CPC-based scaffolds (diameter 5 mm) with interconnected pores. In vitro dissolution studies showed that almost 100% of the drug released within 2 h for all scaffolds. The anticancer cell studies confirmed the effective cell-killing ability of these 5-FU coated CPC scaffolds.	2020	[129]
Magnetite powder	Monetite	In vitro	Magnetic monetite (CaHPO4)-based calcium phosphate cements (CPCs) compositions developed for the hyperthermia treatment of bone tumors. This bioactivity cement composition generated heat in the range of 40–45 °C when an electromagnetic field was applied. The generated heat is enough to kill the tumor cells without destroying healthy cells. The in vitro studies further confirmed that the composition was biocompatible with pre-osteoblast cells.	2020	[270]
Doxorubicin	HA	In vivo	Cement combining amorphous calcium phosphate, folic acid and doxorubicin-loaded particles of carbonated nanocrystalline HA, as a means of local release. Preliminary in vivo data on an invasive osteosarcoma rat model suggest a limiting effect on metastatic events without signs of toxicity.	2021	[271]
Doxorubicin, cisplatin, etoposide, SF2523	HA	In vitro	Chemotherapeutic agents including doxorubicin, cisplatin, etoposide, and SF2523 were mixed with cement. There was a significant decrease in the cell proliferation of ES cells by 48 h post-exposure. There was a synergistic effectiveness of the cement noted when multiple antineoplastic agents were combined.	2023	[272]
Zoledronic acid	HA	In vitro	Methylcellulose/gelatin/calcium phosphate cement-based (CPC) was loaded with zoledronic acid (ZOL) to induce anti-osteoporosis and anticancer properties, and oxide graffene (GO) was incorporated into cement to improve the physical properties of the samples. ZOL- and GO-loaded CPC revealed clinically suitable properties with the controlled release of ZOL, pH value and PO_4_^3−^ ions. In in vitro cell studies, both the inhibitory effects of cement on human breast cancer cell line (MCF-7) cells and proliferative effects on osteoblast cells were observed.	2021	[273]
Quercetin	Brushite	In vitro In vivo	Scaffolds composed of brushite cement (CPC) containing quercetin lipid nanosystems were prepared. In vitro tests proved that the addition of the quercetin–phospholipid complex within nanostructured lipid carriers (QT-NLC) did not deteriorate the properties of CPC: setting time, strength, or porosity. Using a rat femur bone defect animal model, the histological results show that the QT-NLC/CPC had superior bone healing potential.	2023	[274]
Peptide CGRP	HA	In vitro	Strontium (Sr)–calcium phosphate cement (CPC) with chitosan and gene-related peptide (CGRP) was developed. The results show that CGRP/chitosan-Sr-CPC could release CGRP and enhance the proliferation of human umbilical vein endothelial cells (HUVECs) via CGRP receptors, significantly upregulating the expression of the VEGF gene.	2018	[275]
GDF5, BB-1, BMP-2)	Brushite	In vitro	Brushite-forming calcium phosphate cement (CPC) was mixed with stabilizing poly(l-lactide-co-glycolide) acid (PLGA) fibers and bone morphogenetic proteins (GDF5, BB-1, and BMP-2). Considerable proportions of BMP were released from the CPC within 31 days; the presence of PLGA fibers significantly enhanced the BMP release within 14 days. The released BMPs demonstrated bioactivity, in some cases augmented by the addition of 10% PLGA fibers.	2019	[276]
BMP-2	HA	In vivo (clinical)	This study aimed to identify the clinical osteogenic effect of recombinant human bone morphogenetic protein-2 (rhBMP-2) loaded in calcium phosphate cement (rhBMP-2/CPC). The quantity of new bone formation in the experimental group was greater than that in the control group. rhBMP-2/CPC has osteogenic potential.	2022	[277]
Human Adipose Tissue Stem Cells (hASC)	HA	In vitro	Injectable nanocrystalline calcium phosphate cement was found to function as a delivery system of stem cell-laden gelatin fibers. CPC had several vacant channels generated out of the dissolved gelatin. The proliferation and attachment of the cells were observed inside of the channels. The osteogenic differentiation of gelatin fiber-delivered cells was observed.	2022	[278]

## Data Availability

Not applicable.
